# A Case Report: Low Voltage Electric Injuries Culminating in Cardiac Arrest and Direct Lung Injury

**DOI:** 10.7759/cureus.11261

**Published:** 2020-10-30

**Authors:** Filipa Guimarães, João Camões, Ana Mesquita, Ernestina Gomes, Rui Araujo

**Affiliations:** 1 Internal Medicine Department, Unidade Local de Saúde de Matosinhos (ULSM) - Hospital Pedro Hispano, Porto, PRT; 2 Intensive Care Unit, Unidade Local de Saúde de Matosinhos (ULSM) - Hospital Pedro Hispano, Porto, PRT

**Keywords:** high voltage, low voltage, cardiac arrest, lung electric injuries, electrical injuries

## Abstract

Serious electrical injuries are rare but may have life-threatening consequences. Voltage exposure injuries are divided into low voltage injury (LVI) or high voltage injury (HVI). An LVI current can result in severe injury, depending on the length of exposure, the size of the individual, the cross-sectional area in contact with the electrical source, and environmental humidity.

The authors present a 31-year-old male with accidental electrocution with low voltage current and cardiopulmonary arrest. A detailed revision by organs and systems is presented.

LVI is uncommon and can occur with a variety of clinical presentations, rarely presenting with direct lung injury. Early recognition and support are the cornerstones of treatment.

## Introduction

Serious electrical injuries are rare but may have significant life-threatening consequences. Voltage exposure injuries are classically divided into low voltage injury (LVI) or high voltage injury (HVI) using a cutoff value of 1000V, as well as by whether electrical current flows directly through the body versus a thermal injury caused by an electrical flash. However, the correlation between injury severity and the electromotive force is largely affected by various other factors [1]. A low voltage electrical current can result in a severe injury, depending on the length of exposure, the size of the individual, cross-sectional area in contact with the electrical source, and environmental humidity. The authors present a case of accidental electrocution with a low voltage current with cardiopulmonary arrest and lung injury. Written informed consent was taken from the family of the patient.

## Case presentation

**Figure 1:**
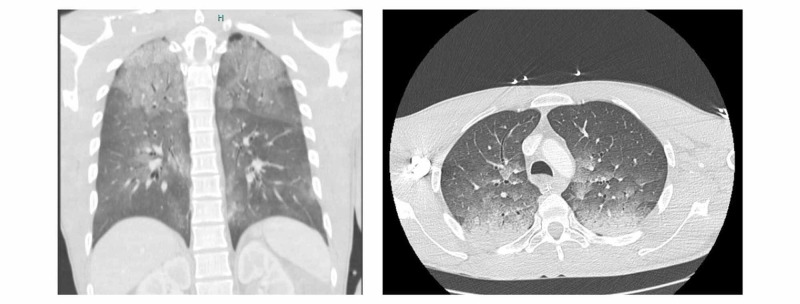
Thoraic CT scan

A 31-year-old male with no known medical problems suffered an LVI due to malfunction of a water cylinder while opening the shower tap, resulting in a cardiopulmonary arrest shortly thereafter. Bystander basic life support (BLS) was promptly initiated and maintained for about 10 minutes before the arrival of the first pre-hospital team. Advanced life support (ALS) was maintained for an additional 20 minutes, with an initial shockable rhythm (ventricular fibrillation). After a total of 30 minutes of cardiopulmonary resuscitation (CPR), recovery of spontaneous circulation (ROSC) was achieved. The patient did not regain consciousness and was sedated and ventilated. Upon arrival at the hospital, blood pressure was 89/60 mmHg; heart rate 80 bpm, and tympanic temperature 35ºC. Glasgow Coma Scale (GCS) was 3/10 with symmetric pupils. The blood glucose level was 261 g/dL. No entrance or exit site wounds were identified. Initial blood gas analysis revealed mixed acidosis (pH 7.03, partial pressure of carbon dioxide (pCO2) 62 mmol/L, bicarbonate (HCO3) 16.4 mmol/L, lactate 8.4 mmol/L), which was then corrected with ventilator adjustments and fluid resuscitation. Additional tests revealed an acute kidney injury (Cr 1.6 mg/dL, Urea 40 mg/dL), hyperphosphatemia (11.4mg/dL), rhabdomyolysis (creatine kinase (CK) 406U/L, myoglobin 6241 ng/mL), cytolysis (aspartate aminotransferase (AST) 102 U/L, alanine transaminase (ALT) 101 U/L); elevated troponin (750 ng/L for a normal of < 34.5ng/L), and a negative screening for drug abuse. Thyroid function and other serum electrolytes were normal; transthoracic echocardiography and electrocardiogram (EKG) had no major changes, angio-computed tomography (CT) showed no evidence of pulmonary thromboembolism, but ground-glass opacities of both lungs were described, mainly on the upper lobes, as well as a consolidative image on the right lower lobe (Figure 1).

The patient was admitted to the intensive care unit (ICU). Target temperature management protocol and supportive measures were initiated for a period of 24 hours. The patient had a positive outcome and was discharged 24 days after admission.

A detailed description of the evolution is described hereunder.

Neurologic

Cerebral CT at admission was normal; the target temperature control protocol was maintained for 24 hours. The patient gradually recovered to a GCS of 11. No focal motor deficits were found. Electroencephalogram (EEG) excluded paroxysmal activity and complementary magnetic resonance imaging (MRI) revealed hyperintense areas suggesting cytotoxic edema. 

Cardiovascular

Mean arterial pressure (MAP) was >75 mmHg with a transitory need for aminergic support and fluid resuscitation. No arrhythmias were registered. Troponin monitoring showed a maximum of 33.000 ng/L (12 hours after admission) with posterior normalization; EKG had some unspecific changes but was normalized in a few days. Lactates normalized and the transthoracic echocardiogram was normal.

Pulmonary

The patient remained mechanically ventilated for five days, with minimal respiratory insufficiency (minimum partial pressure of oxygen (pO2) to fraction of inspired oxygen (FiO2) ratio of 250 with protective mechanical ventilation and pCO2 was successfully titred to values of 36-40 mmHg). On the first day of admission, the patient was started on antibiotics (amoxicillin-clavulanate) assuming aspiration due to the constellation of fever (39ºC after targeted temperature management (TTM) was stopped), productive sputum, and consolidation pattern on lower thorax CT scan. More interestingly, ground-glass opacities of both lungs at admission were also present. Successful extubation was possible on the fifth day of ICU.

Renal

Acute renal injury with a maximum serum creatinine of 1.6 g/dL, resolved. Urine output maintained values > 200 mL/kg

Muscle and skin

Rhabdomyolysis resolved in 72 hours (creatine kinase (CK) maximum of 3700 U/L; myoglobin maximum of 6241 U/L). No burns or skin lesion suggestive of entrance or exit wounds were found.

Hepatic

No liver dysfunction was detected. There was transient AST and ALT elevation, with a maximum of 3x the normal values at admission and posterior normalization.

The patient was discharged to a rehabilitation center, where he showed motor and cognitive improvement over three months. At the follow-up evaluation, he demonstrated the ability to perform several daily base activities, such as feeding, dressing, and bathing, although his ability to perform highly cognitive-demanding procedures was compromised.

## Discussion

LVI is uncommon and can occur with a wide variety of clinical presentations, potentially involving all organs and systems.

Nervous system

Since electricity favors the path of least resistance, such as vessels and central nervous system (CNS) tissue, it is no surprise that neurological complications after LVI are the most frequently reported. Besides, electricity induces myelin damage via direct influences and via vascular endothelial damage and results in impaired nerve blood supply [2]. Our patient did develop generalized muscle weakness. Sensory abnormalities were difficult to evaluate.

Encephalopathic syndromes are prone to occur in patients following electrical injury and neuropsychological disorders are present in up to 50% of these patients [3]. In the present case, lesions suggesting cytotoxic edema were seen on MRI. Although it could result from LVI, it is much more common after hypoxic injury due to cardiopulmonary arrest.

Heart

The true incidence of cardiac involvement after an electrical accident has been inconsistently reported, although rhythm disturbances are the most frequently outlined in this case [4]. In one recent study, cardiac arrhythmias were observed in 14% of patients admitted after electrical injury. More interestingly, the presence of ventricular tachycardia/ventricular fibrillation was reported in three patients, one of whom was injured after a low voltage accident, and asystole/pulseless electrical activity was only present in patients who suffered HVI [1]. Exposure to HVI is more frequently associated with ventricular asystole than LVI [1,4]. This patient was previously healthy, and we assumed that the initial ventricular fibrillation period was directly induced by voltage. Moreover, his re-evaluation EKGs and cardiac monitoring were always within the normality, which is consistent with a unique event in time induced by external aggression. Significant troponin elevation observed in our patient was likely due to prolonged CPR maneuvers [5].

Structural damage and the impaired function of the heart following electrical damage have also been recorded [4]. Fortunately, in this case, ejection fraction was preserved and subsequent EKGs showed a complete reverse of initial changes.

Lung

Voltage-induced lung injury has been demonstrated in case reports. Although the real incidence of direct lung damage is unclear, it is a rare phenomenon [6]. Two distinct patterns are described in the literature: pneumomediastinum [7] and parenchymal/interstitium lesions that vary from non-cardiogenic edema to consolidation [6,8]. This patient presented with a thorax CT scan that showed bilateral ground-glass opacities.

Other case reports published also present patients with ground-glass opacities [8] after electrical injury but only Truong and associates [6] described a case caused by LVI.

We concluded that these bilateral, upper changes may correspond to direct electrical lung injury since we assumed electrical arch throughout the hands, arms, and upper chest, and other contributions such as CPR-induced injuries were considered less probable due to the exclusively superior/apical location; on the other hand, the consolidation described in the right lower seems to be associated with aspiration of gastric content.

Muscle and skin

Electrical injury is a known cause of severe rhabdomyolysis. Elevated levels of serum CK and several other muscle fiber components are frequent and the relation of CK elevation with the amount of burned body surface area is still a matter of debate. As expected, our patient showed maximum levels of myoglobin at admission and a posterior elevation of CK, which were both normalized at the time of ICU discharge. These findings are in line with other reports [1].

One intriguing point in the first initial diagnostic workup was the total absence of entry or exit wounds. However, in a large report of 220 electrical fatalities, more than 40% of patients suffering LVI didn't have apparent burn injuries [9]. Our patient had an LVI during a shower, which could explain the absence of lesions. Wet skin offers less resistance to the passage of an electric current and a more diffuse entry area and, therefore, leads to less probability of a burn mark at the entry site.

Kidney

Acute kidney injury in the setting of electrical injury can occur due to direct visceral damage [10] or due to systemic rhabdomyolysis [1]. The pathophysiology of AKI includes hypovolemia, myoglobinuria, and metabolic acidosis. In this setting, fluid management was the only approach necessary.

Liver

Visceral lesions are rare but potentially severe. Electrical burns itself can damage the tissues by heat or by electrical current, causing coagulative necrosis and cell membrane rupture, but the true pathophysiology of electrical injury of internal organs is still unclear. There are several reports on colon and intestine affection. It is less frequent than the involvement of other organs such as the liver and kidney [10].

In the present case, no other abdominal organ injuries were documented apart from the kidney and liver. Our patient presented with mild cytolysis, with no liver dysfunction or cholestasis, and the evolution was positive, with no direct measures to this problem.

## Conclusions

LVI is uncommon and can occur with a wide variety of clinical presentations. Early recognition and organ support are the cornerstones of treatment. The authors emphasize the presence of ventricular fibrillation in cardiac arrest due to LVI, the rare presentation of pulmonary ground-glass findings, and the absence of entry/exit wounds. These were the most remarkable and challenging findings and should be exhaustively searched for in these patients.
